# Unexpected complications of vasospastic coronary artery disease and its successful management

**DOI:** 10.15171/jcvtr.2019.28

**Published:** 2019-05-18

**Authors:** Gülay Gök, Tufan Çinar

**Affiliations:** ^1^Medipol University School of Medicine, Department of Cardiology, Istanbul, Turkey; ^2^Health Science University, Sultan Abdülhamid Han Training and Research Hospital, Department of Cardiology, Istanbul, Turkey

**Keywords:** Vasospastic Coronary Artery Disease, Myocardial Infarction, Percutaneous Coronary Intervention, Complication

## Abstract

Vasospastic coronary artery disease (CAD) usually occurs during the percutaneous interventions and responds to conventional medical treatment. However, in rare conditions, it may be resistant to medical treatment, resulting in lethal complications, including acute myocardial infarction, ventricular arrhythmia, cardiopulmonary arrest, cardiogenic shock, and acute pulmonary edema. In this case report, a 44-year-old woman was admitted to the hospital with a diagnosis of non-ST-segment elevation myocardial infarction. During a diagnostic coronary angiography and in-hospital stays, multiple catastrophic complications due to vasospastic CAD occurred, and we were able to demonstrate a successful management strategy of these complications.

## Introduction

Vasospastic coronary artery disease (CAD) is thought to result from endothelin dysfunction, which impairs vasodilatation.^1^ Vasospastic CAD usually occurs during the percutaneous interventions and responds to conventional medical treatment. However, in rare conditions, it may involve multiple vessels, and it may be resistant to medical treatment, resulting in lethal complications, including acute myocardial infarction, ventricular arrhythmia, cardiopulmonary arrest, cardiogenic shock, and acute pulmonary edema.^2,3^ In this paper, a rare case of vasospastic CAD with multiple catastrophic complications and its successful management strategy is reported.

## Case Report

**Figure 1:**
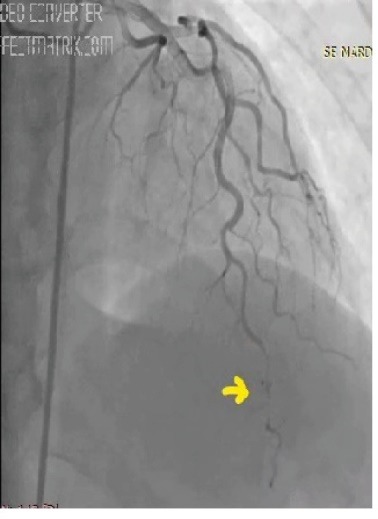
Coronary angiogram of left coronary artery in posterior-anterior cranial view showing a severe spasm of the distal left anterior descending artery and obtuse marginal artery (arrow).

**Figure 2:**
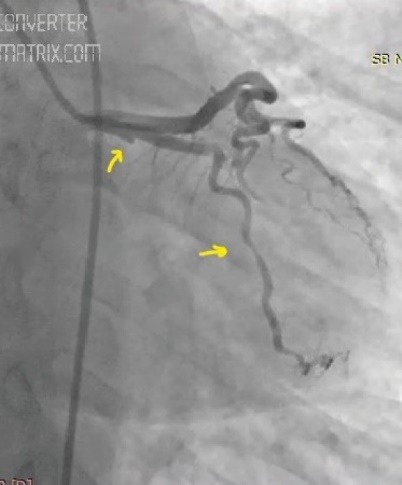
Right anterior oblique caudal view showing an occurrence of no-reflow in the circumflex artery (arrows).

**Figure 3:**
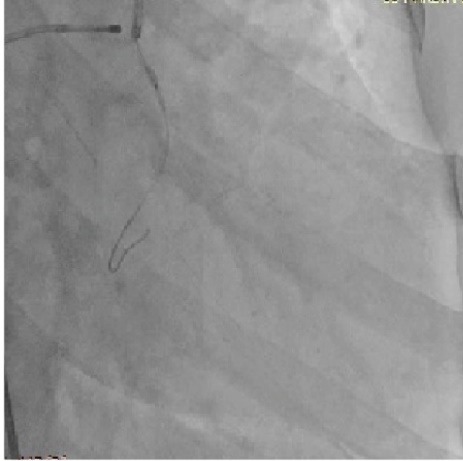
The mid-portion of the circumflex artery was dilated using a 1.5x9 mm balloon.

**Figure 4:**
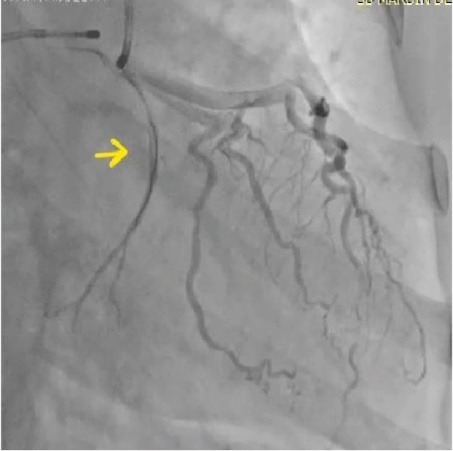
After dilatation of the mid-portion of the circumflex artery with a balloon, the spasm was relieved (arrow).

**Figure 5:**
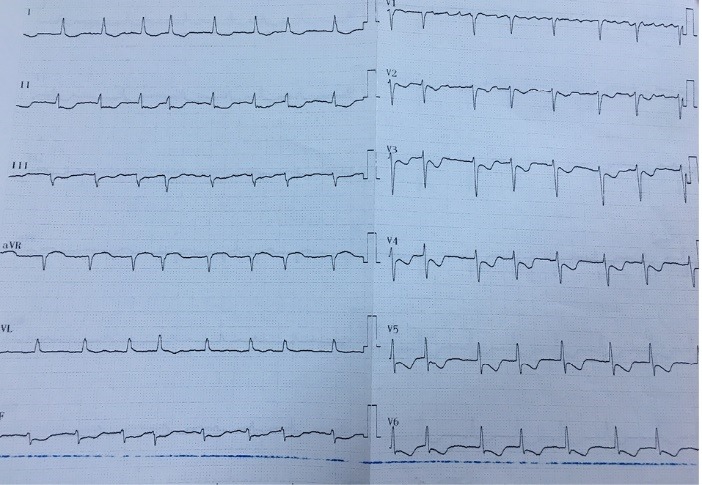
An electrocardiogram obtained after post-resuscitation showing ST depression on the anterior leads.

A 44-year-old woman was admitted to the hospital, complaining of intermittent chest pain that began two weeks earlier. The chest pain’s strength gradually worsened the last few days. In her medical history, the patient had no history of hypertension, diabetes mellitus, smoking, dyslipidemia, or family history of cardiovascular disease. Two years ago, she had a history of coronary angiography, which revealed a significant occlusion of the mid-segment of the left anterior descending (LAD). At that time, a stent implantation was recommended; however, she refused it and was discharged from the hospital with medical treatment. Upon admission this time, the patient’s physical examination was normal with a blood pressure of 125/80 mm/Hg and heart rate of 72 beats/min. Her electrocardiography (ECG) showed poor R-wave progression on the anterior leads. The laboratory analysis demonstrated that cardiac enzymes levels were above the normal limits. She was diagnosed with non-ST-segment elevation myocardial infarction, and medical treatment commenced. The patient’s treatment consisted of metoprolol (50 mg/d), isosorbide mononitrate ER (60 mg/d), atorvastatin (40 mg/d), enoxaparin (0.1 cc/kg), acetyl salic acid (300 mg/d), and clopidogrel (75 mg/d)]. Transthoracic echocardiography was performed providing a left ventricular ejection fraction of 30%-35%, global left ventricular hypokinesia, and mild mitral regurgitation. Therefore, we could not consider of calcium channel antagonists treatment. All medications were given at the maximum dosages the patient could tolerate. A coronary angiography via femoral approach was performed using a 6 French Judkins left 4 diagnostic catheter; it revealed a severe spasm of the distal LAD and obtuse marginal artery, which was not present in the previous catheterization (Figure 1). On the fifth injection to the left coronary artery, no-reflow occurred in the circumflex artery (CX) beyond its proximal part (Figure 2). At that time, the patient experienced severe chest pain and developed a complete atrioventricular block with a heart rate of 35 beats/min. Her blood pressure fell to 73/50 mm/Hg. Despite profound hypotension, intracoronary 500 μg of nitroglycerin and 1 mg of atropine were administered. However, an intracoronary injection of nitroglycerin was insufficient in relieving the spasm, so a percutaneous coronary intervention was planned. Initially, a temporary transvenous pacemaker was inserted in the right ventricle. Then, a 0.014-inch floppy was advanced through the CX. The mid-portion of the CX was dilated with a 1.5x9 mm balloon (Figure 3). The dilatation with the balloon eventually relieved the spasm and improved the blood pressure (Figure 4). After hemodynamic stabilization, she was transferred to the coronary care unit and was closely observed under the medical treatment. On the third day of hospital admission, she developed a pulseless ventricular fibrillation. She was defibrillated and needed endotracheal intubation. After 5 minutes of cardiopulmonary resuscitation, she was successfully returned to sinus rhythm. Her post-resuscitation ECG showed ST depression on the anterior leads (Figure 5). A continuous low dose of intravenous nitroglycerin was given, the ST segment depression on the anterior leads was resolved, and she was successfully extubated the second day. An implantable cardioverter defibrillator (ICD) was implanted due to ventricular fibrillation. Thereafter, she was discharged from the hospital with medical treatment, including, metoprolol (50 mg/d), isosorbide mononitrate ER (60 mg/d), atorvastatin (40 mg/d), aspirin (100 mg/d), and clopidogrel (75 mg/d). During a follow-up period of 30 months, the patient was without symptoms.

## Discussion

The true incidence of vasospastic CAD has been unknown due to diagnostic challenges, but the incidence is higher in young populations, females, and Japanese people. The underlying pathophysiological mechanism of coronary artery spasm is not yet known. However, it is thought to be mainly because of endothelial dysfunction, which impairs vasodilatation. Cocaine abuse, smoking history, oxidative stress, inflammation, and genetic background may predispose one to vasospastic CAD.^4^ However, in this case report, the patient had no history of predisposing factors for vasospastic CAD.

In this case, a clinical diagnosis of vasospastic CAD was made after observing significant stenosis in multiple and different locations compared to previous catheterization. Multisite and multi-vessel involvement without atherosclerotic lesions was a predictor of vasospastic CAD. During angiography, vasospastic CAD may be provoked by a catheter, guide wire insertion, balloon dilatation, and stent implantation or it may be a spontaneous phenomenon.^5,6^ In this case, the spontaneous induction of the CX spasm was predicted during diagnostic coronary angiography since the localization of the spasm was far away from the tip of the catheter. The localization of the spasm may be focal, multiple, or may include the entire coronary system.^5^ Patients with diffuse and multiple spasms are generally refractory to conventional medical therapy and have worse outcomes.^7^ Vasospastic CAD typically responds to coronary vasodilators; however, it can be refractory to medical therapy and may require balloon dilatation or stent implantation. In this case, the failure of intracoronary nitroglycerin treatment led us to opt for percutaneous coronary intervention.

The medical treatment of vasospastic CAD is calcium channel antagonists and nitrates because they reduce and resolve spasm attacks. The calcium channel antagonists suppress calcium ion (Ca2+) inflow into the vascular smooth muscle, and the nitrates metabolize to nitrite oxide, resulting in the relaxation of vascular smooth muscle and vasodilation. Because the patient had a low left ventricular ejection fraction, we could not give a calcium channel antagonists therapy. In the treatment of vasospastic CAD, there are still uncertainties about how to manage these patients and when to consider the use of ICD for primary prevention.^4^ When associated with multi-vessel involvement, vasospastic CAD has poor prognosis, as in this case.^8^ The previous studies strongly recommend ICD implantation in patients with vasospastic CAD for secondary prevention of sudden cardiac death, emphasizing the high recurrence risk of ventricular arrhythmias in those patients despite optimal medical treatment.^9,10^

## Conclusion

Vasospastic CAD may have catastrophic results and it may be refractory to conventional medical therapy. These patients usually have poor prognosis and may require the consideration of ICD implantation.

## Ethical approval

An informed consent was taken from the patient for publishing this case report.

## Conflict of interest

All authors declare that they do not have conflict of interests.

## Funding

The authors did not receive any specific funding for this work.
